# Protective effect of TM6 on LPS-induced acute lung injury in mice

**DOI:** 10.1038/s41598-017-00551-8

**Published:** 2017-04-03

**Authors:** Xiaoyu Hu, Yuan Tian, Shihui Qu, Yongguo Cao, Shumin Li, Wenlong Zhang, Zecai Zhang, Naisheng Zhang, Yunhe Fu

**Affiliations:** Department of Clinical Veterinary Medicine, College of Veterinary Medicine, Jilin University, Changchun, Jilin Province 130062 P.R. China

## Abstract

Acute lung injury (ALI) is an acute failure of the respiratory system for which effective treatment is urgently necessary. Previous studies found that several peptides potently inhibited the production of cytokines induced by lipopolysaccharide (LPS). In this study, we synthetized a cell-permeable TIR domain-derived decoy peptide (TM6) and examined its substance for the ability to inhibit TLR signaling in the model of ALI induced by LPS. We demonstrated that TM6 (2.5, 5 and 10 nmol/g) alleviated the histological changes in the lung tissues as well as myeloperoxtidase (MPO) activity, lung W/D ratio, the production of TNF-α, IL-1β and IL-6 induced by LPS. Furthermore, the numbers of total cells, neutrophils and macrophages in the BALF were suppressed by TM6. *In vitro*, TM6 (5, 10 and 20 µM) inhibited the production of TNF-α, IL-1β and IL-6 in LPS-stimulated alveolar macrophages. Moreover, the activation of Nuclear factor-kappaB (NF-κB) and Mitogen activated protein kinases (MAPK) signaling pathways induced by LPS were also inhibited by TM6. Collectively, our results suggested that TM6 was an effective inhibitor of ALI induced by LPS, and this peptide may very well serve as a future treatment for ALI.

## Introduction

Acute lung injury (ALI) has been recognized as a life-threatening disease, which is usually caused by pneumonia, sepsis, shock and aspiration^1, 2^. The major characteristic of ALI, - which is the key factor leading to Acute Respiratory Distress Syndrome (ARDS), is the neutrophil accumulation, increasing vascular permeability and arousing pulmonary edema^3, 4^. It is a common clinical problem that presents high morbidity and mortality worldwide^5^. Although there are some medicine to treat ALI patients, the high mortality rate of ALI even reaches a rate of 30–50%^6^. Therefore, to cure ALI, specific and effective treatments are urgently needed. Lipopolysaccharide (LPS) is widely accepted to establish ALI models. LPS has the ability to induce the releases of numerous inflammatory mediators, including TNF-α, IL-1β, IL-6, NO and superoxide anions^7–9^. Additionally, LPS could activate Toll like receptor 4 (TLR4), which subsequently induces the activation of NF-κB and MAPK signaling pathways^10, 11^.

Activation of TLR4 induces the accumulation of TIR domain-containing adapter protein, including MyD88, TRIAP, TRIF, and TRAM. Upon TLR4 activated, TRAM was served as a bridging adapter directly to recruit TRIF and render the production of cytokines. It has been suggested in some study that the recruitment of adapter proteins is through the interaction of TIR-TIR domains. The TIR domain of TLR4 is consists of five-stranded parallel β-sheet and five α-helices^12, 13^. We synthetized some TRAM TIR domain-derived decoy peptides and indicated that TM6 or TR6 inhibited the development of mastitis induced by LPS in mice^14, 15^. It has been reported that certain decoy peptides have been demonstrated modest inhibition to several the signaling outcomes, suggesting the possibility of pathway-specific interaction surfaces^13^. Pfalzraff A found that synthetic antimicrobial and LPS-neutralising peptides inhibited the production of inflammatory cytokines through inhibiting NF-κB and MAPK signaling pathway^16^. Other studies have confirmed that TM6 was capable to efficiently prevent the LPS-induced TLR4/MyD88 association and significantly decrease circulating levels of both cytokines^17^. The TM6 (sequence: “N”-RQIKIWFQNRRMKWK and,-KENFLRDTWCNFQFY-“C”) is derived from the third helical region of TIR domains of TRAM. TM6 exhibited anti-inflammatory effects through the blocking of the docking site of its prototype function protein, which lead to the inhibition of the interaction of TRAM with downstream adaptor molecular. The therapeutic potential of TM6 has ever been mentioned in individual papers^14, 17^. However, the effects of TM6 on LPS-induced ALI remains unclear. In the present study, we investigated the therapeutic effects and mechanisms of TM6 on LPS-induced ALI both *in vivo* and *in vitro*.

## Results

### The effect of TM6 on histopathology changes in lung tissues

**Figure 1:**
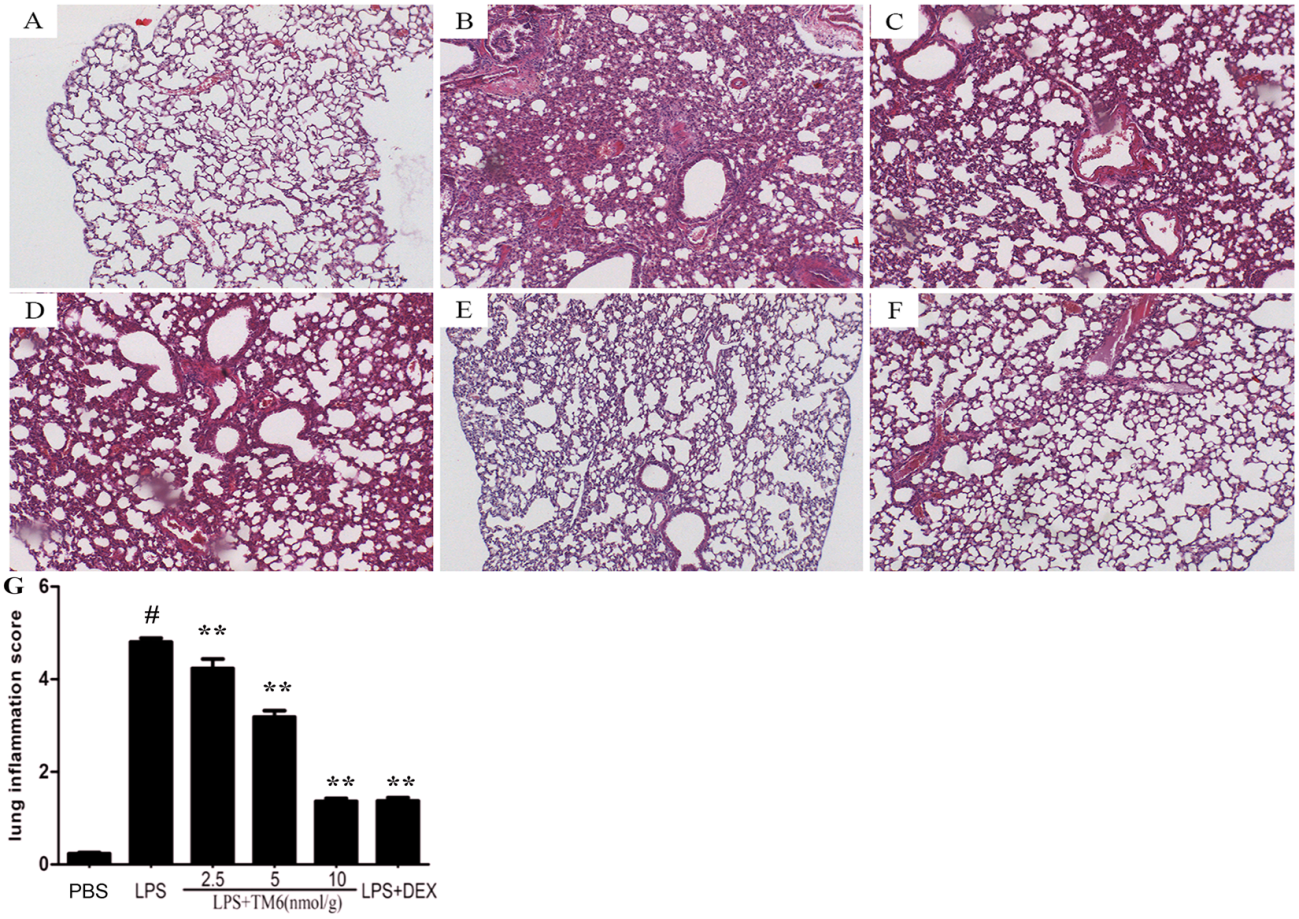
The effect of TM6 on lung tissue histopathological changes. Histopathologic sections of lung tissues (H and E, ×100). (**A**) Lung tissues from control, (**B**) LPS, (**C**) LPS + TM6 (2.5 nmol/g), (**D**) LPS + TM6 (5 nmol/g), (**E**) LPS + TM6 (10 nmol/g), and (**F**) LPS + DEX (5 mg/kg) mice, (**G**) the pathological scores was evaluate during ALI.

7 h after LPS stimulation, histopathology changes were determined by H&E staining. The lung tissues of LPS group showed several obvious inflammatory changes, such as lung edema, pulmonary congestion, alveolar hemorrhage, thickening of the alveolar wall and areas of inflammatory infiltration (Fig. 1B). Treatment with TM6 (2.5, 5 and 10 nmol/g) and DEX (5 mg/kg) markedly alleviated the histopathology changes induced by LPS (Fig. 1C,D,E,F). In addition, the scores was evaluated to determine the thickness of alveolar walls and epithelium, the increased number of infiltration cells in perbronchial, and perivascular cuff area. As it is shown in Fig. 1G, the mean pathological score was significantly increased after LPS administration compared with control group (0.30 ± 0.003 vs 4.86 ± 0.08). However, the pathological scores were reduced by treatment of TM6 (4.15 ± 0.09, 3.72 ± 0.06, 1.81 ± 0.04, 1.84 ± 0.06 vs 4.86 ± 0.08) in a dose-dependent manner.

### The effect of TM6 on wet/dry lung ratios

**Figure 2:**
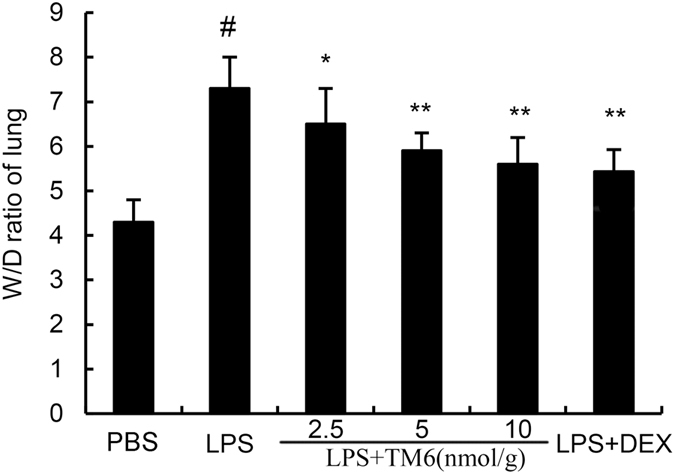
The effect of TM6 on lung wet/dry radio. Mice were pretreated with TM6 (2.5, 5 and 10 nmol/g) and DEX (5 mg/kg) 1 h prior to an i.n. administration of LPS. The lung wet/dry ratio was determined 7 h after LPS challenge. The values presented are the means ± SEM (n = 6). ^#^P < 0.01 is significantly different from the control group; ^*^P < 0.05 and ^**^P < 0.01 are significantly different from the LPS group.

Lung W/D ratios were evaluated 7 h after LPS stimulation. The lung W/D ratio was markedly higher after LPS administration compared to the control group. However, TM6 (2.5, 5 and 10 nmol/g) significantly reduced the lung W/D ratio induced by LPS. In addition, DEX (5 mg/kg) also reduced the Lung W/D ratios after LPS stimulation (Fig. 2).

### The effect of TM6 on inflammatory cell counts in the Bronchial Alveolar Lavage Fluid (BALF)

**Figure 3:**
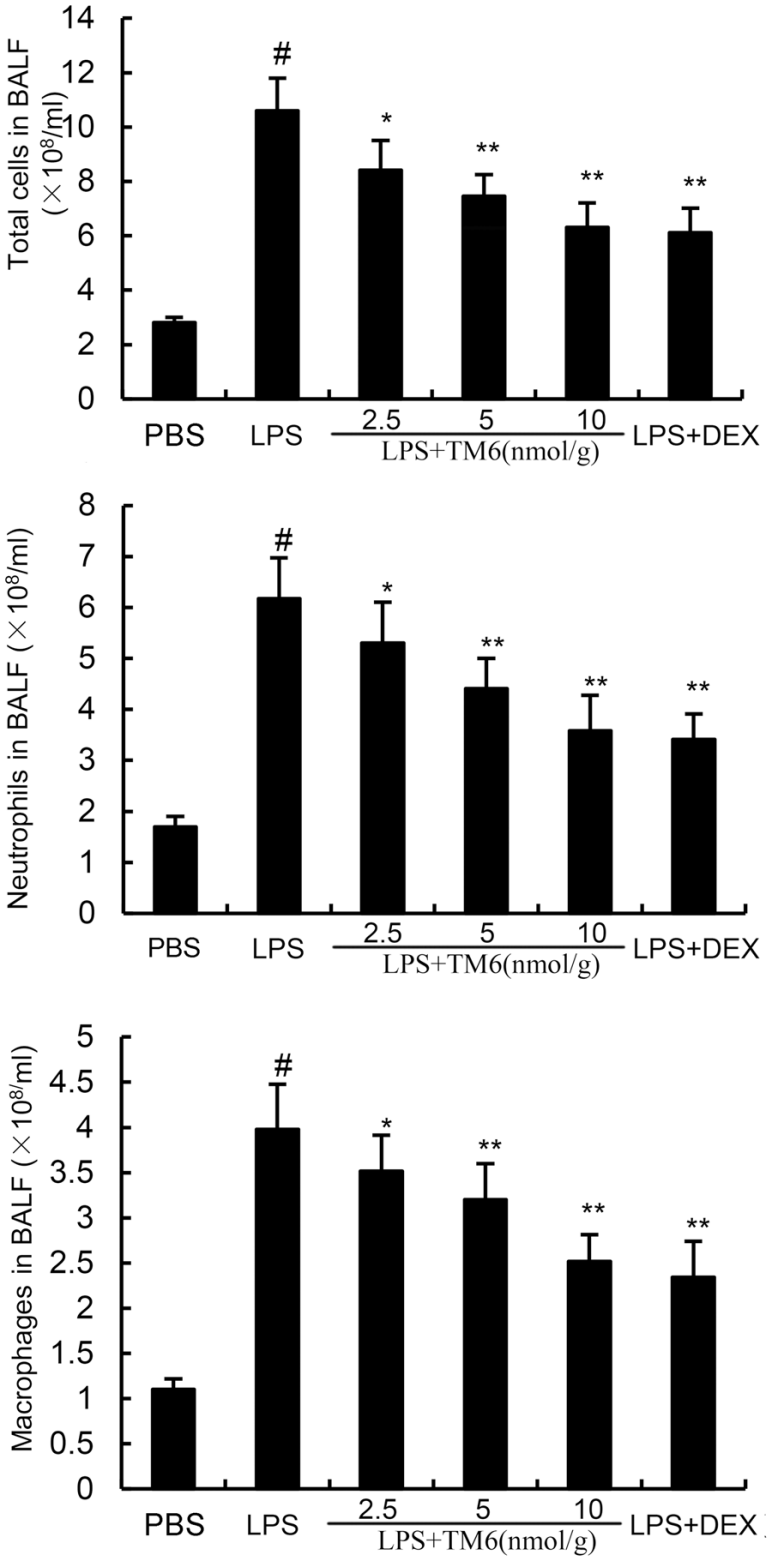
The effects of TM6 on the number of total cells, neutrophils, and macrophages in the BALF. Mice were given an intraperitoneal injection of TM6 (2.5, 5 and 10 nmol/g) and DEX (5 mg/kg) 1 h prior to an i.n. administration of LPS. BALF was collected 7 h after LPS administration to measure the number of total cells (**a**), neutrophils (**b**), and macrophages (**c**). The values presented are the means ± SEM (n = 6). ^#^P < 0.01 is significantly different from the control group; ^*^P < 0.05 and ^**^P < 0.01 are significantly different from the LPS group.

Seven hours after LPS stimulation, the numbers of total cells, neutrophils, and macrophages in BALF were measured. The results showed that stimulation with LPS caused a markedly increase in total cells compared with control group (10.93 ± 0.18 vs 2.51 ± 0.06 × 10^8^/ml), and macrophages (4.02 ± 0.12 vs 1.16 ± 0.03 × 10^8^/ml) and neutrophil production (6.17 ± 0.20 vs 1.82 ± 0.08 × 10^8^/ml). However, treatment of TM6 (2.5, 5 and 10 nmol/g) does-dependently suppressed the numbers of total cells (8.82 ± 0.11, 7.92 ± 0.09, 6.21 ± 0.10 × 10^8^/ml), neutrophils (5.52 ± 0.18, 4.49 ± 0.15, 3.82 ± 0.17 × 10^8^/ml) and macrophages (3.61 ± 0.13, 3.38 ± 0.14, 2.33 ± 0.08 × 10^8^/ml) induced by LPS (Fig. 3). DEX also reduced the total cells (6.02 ± 0.10 × 10^8^/ml), neutrophils (3.72 ± 0.13 × 10^8^/ml), and macrophages (2.31 ± 0.13 × 10^8^/ml) production compared with LPS group (Fig. 3).

### The effect of TM6 on the production of cytokines in BALF

**Figure 4:**
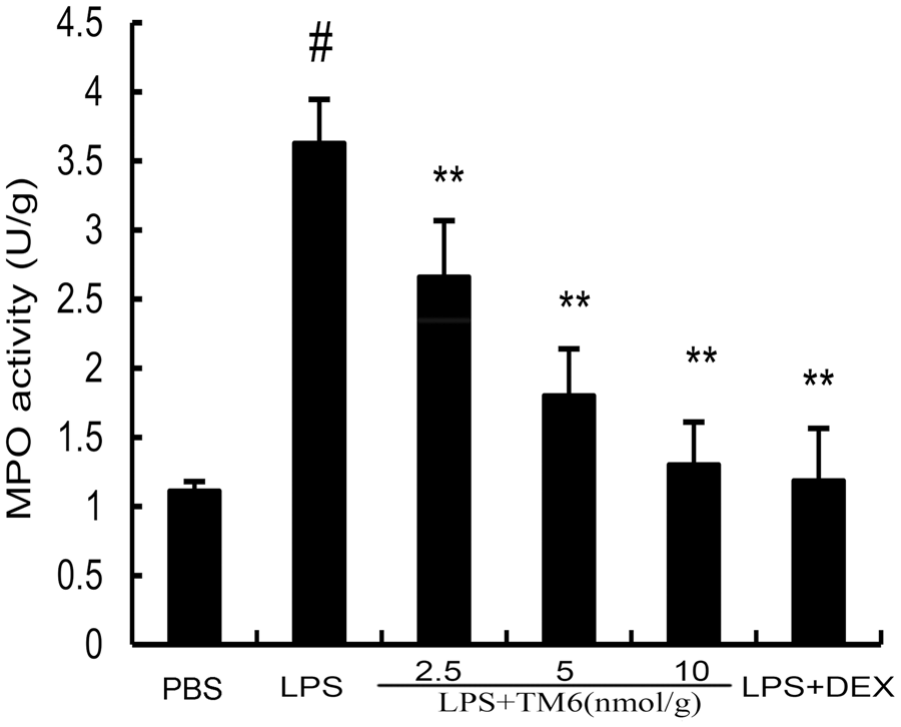
The effect of TM6 on cytokines production in BALF. Mice were given an intraperitoneal injection of TM6 (2.5, 5 and 10 nmol/g) and DEX (5 mg/kg) 1 h prior to an i.n. administration of LPS. BALF was collected 7 h following LPS challenge to analyze the inflammatory cytokines TNF-α, IL-1β, and IL-6. The values presented are means ± SEM (n = 6). ^#^ *P* < 0.01 is significantly different from the control group; ^*^ *P* < 0.05 and ^**^ *P* < 0.01 are significantly different from the LPS group.

To illustrate the effect of TM6 on inflammatory cytokines production, we tested the levels of TNF-α, IL-1β and IL-6 by ELISA. 7 h after LPS stimulation, an evident elevation of TNF-α (2.48 ± 0.08 vs 0.15 ± 0.02 ng/ml), IL-1β (1.81 ± 0.06 vs 0.11 ± 0.01 ng/ml) and IL-6 (1.62 ± 0.05 vs 0.09 ± 0.007 ng/ml) in BALF of LPS group were observed when compared with control group. The levels of TNF-α (1.87 ± 0.09, 1.12 ± 0.05, 0.70 ± 0.02, 0.51 ± 0.04 vs 2.48 ± 0.08 ng/ml), IL-1β (1.43 ± 0.06, 1.13 ± 0.02, 0.72 ± 0.03, 0.66 ± 0.02 vs 1.81 ± 0.06 ng/ml) and IL-6 (1.42 ± 0.07, 1.01 ± 0.04, 0.62 ± 0.018, 0.62 ± 0.019 vs 1.62 ± 0.05 ng/ml) in TM6 (2.5, 5, and 10 nmol/g) or DEX (5 mg/kg) groups decreased significantly when compared with the LPS group (Fig. 4).

### The effect of TM6 on myeloperoxtidase (MPO) activity

**Figure 5:**
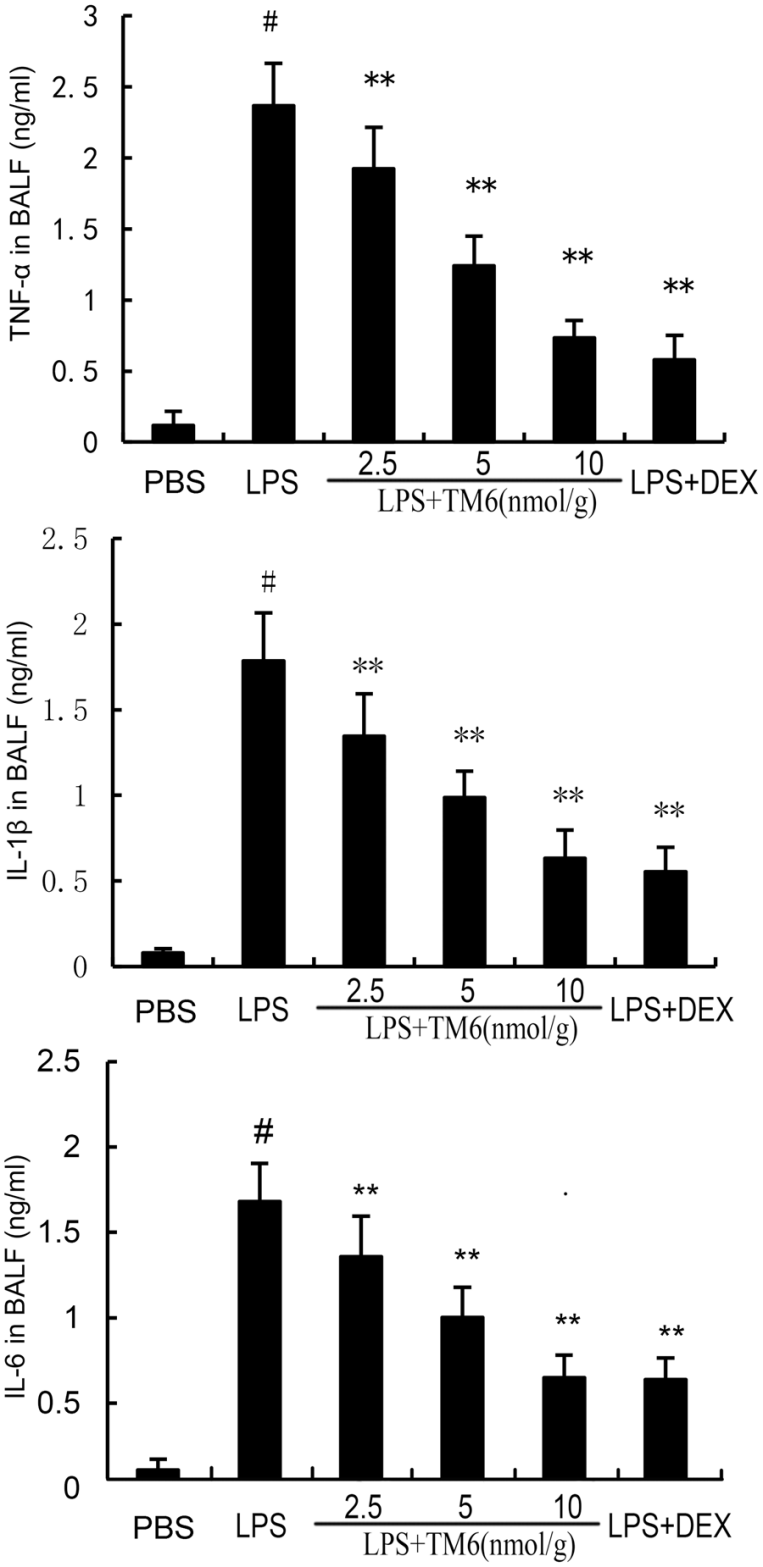
The effect of TM6 on MPO activity. MPO activity was determined 7 h after LPS administration. The values presented are the means ± SEM. (n = 6) ^#^ *P* < 0.01 is significantly different from the control group; ^*^ *P* < 0.05 and ^**^ *P* < 0.01 are significantly different from the LPS group.

MPO activity served as a marker of the development of LPS induced ALI. MPO activity was evaluated 7 h after LPS stimulation. The results showed that lung MPO activity was up-regulated after LPS administration compared with control group. TM6 (2.5, 5, and 10 nmol/g) and DEX (5 mg/kg) administration prevented elevated MPO activity after LPS administration (Fig. 5).

### The effect of TM6 on cell viability

**Figure 6:**
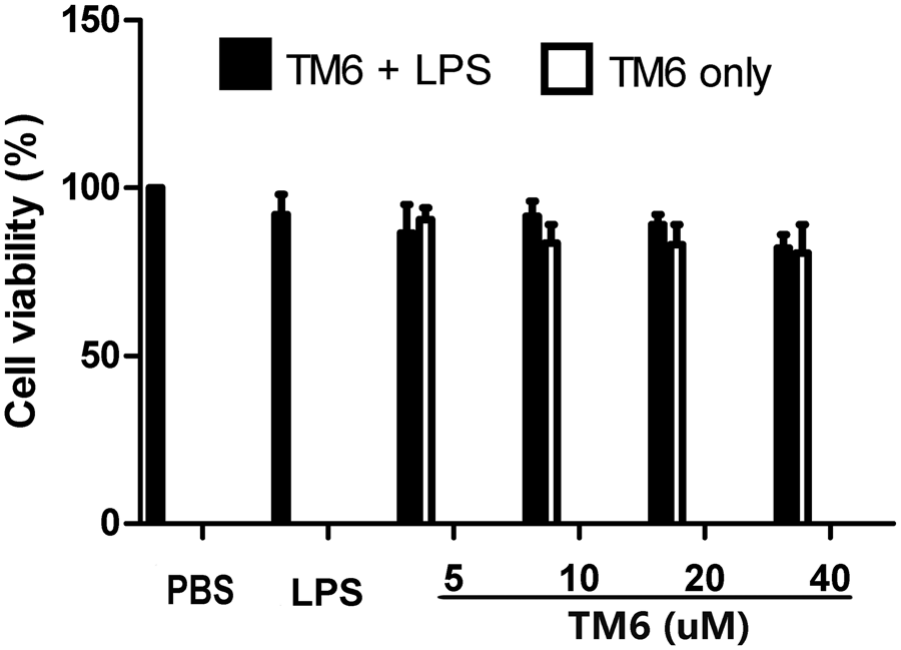
The effect of TM6 on cell viability. Alveolar macrophages extract from mice were treatment with different concentrations of TM6 (0–40 µM) in the absence or presence LPS (1 µg/ml) for 24 hours. The cells viability was tested by MTT assay. The values presented are means ± SEM (n = 6). ^#^ *P* < 0.01 is significantly different from the control group; ^*^ *P* < 0.05 and ^**^ *P* < 0.01 are significantly different from the LPS group.

MTT assay was used to measure the effect of TM6 on the viability of alveolar macrophages. The results showed that TM6 did not affect alveolar macrophages viability at concentrations with 5, 10 and 20 µM (Fig. 6). It suggests that TM6 at the concentrations of 5, 10 and 20 µM did not have any cytotoxic effects on alveolar macrophages.

### The effect of TM6 on inflammatory cytokines production in alveolar macrophages

**Figure 7:**
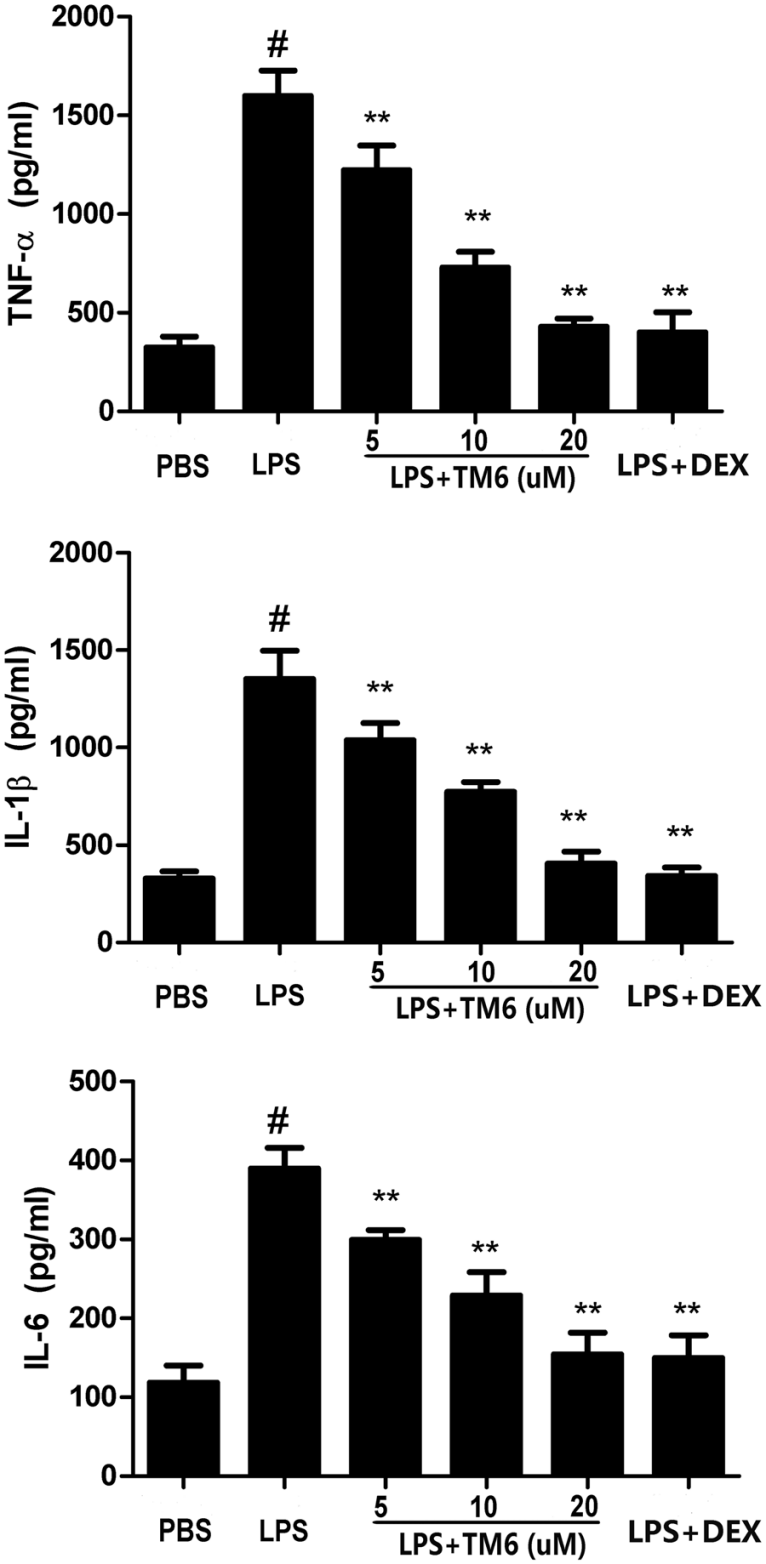
The effect of TM6 on inflammatory cytokines production in alveolar macrophages. Alveolar macrophages were treated with TM6 (5, 10 and 20 µM) and DEX (1 mM) 1 hour prior to stimulation with LPS. 24 hours later, the suspension was collected to test the levels of TNF-α, IL-1β, and IL-6. The values presented are means ± SEM (n = 6). ^#^ *P* < 0.01 is significantly different from the control group; ^*^ *P* < 0.05 and ^**^ *P* < 0.01 are significantly different from the LPS group.

Alveolar macrophages were treated with TM6 (5, 10 and 20 µM) and DEX (1 mM) 1 hour before LPS treatment. 24 hours later, the inflammatory cytokines TNF-α, IL-1β and IL-6 in the supernatant of alveolar macrophages were tested by ELISA. As shown in Fig. 7, after LPS treatment, the production of TNF-α, IL-1β and IL-6 were significantly increased compared with control group. However, TM6 dose-dependently inhibited the production of TNF-α, IL-1β and IL-6 after stimulation with LPS in alveolar macrophages.

### The effect of TM6 on NF-κB and MAPK signaling pathways expression

**Figure 8:**
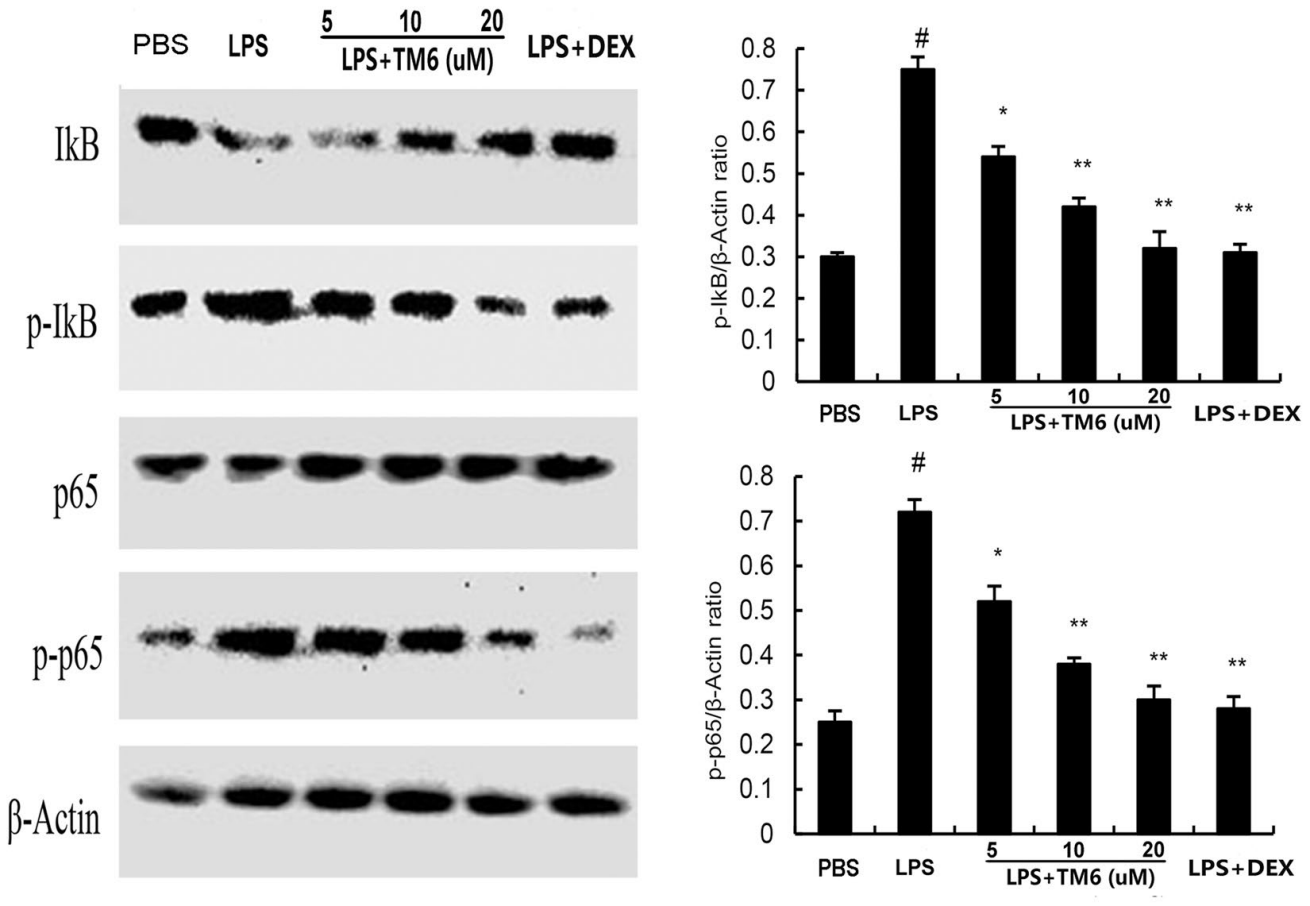
The effect of TM6 on NF-κB pathway. The effects of TM6 on the expression of the NF-κB pathway that was induced by LPS. Alveolar macrophages were pretreated with TM6 (5, 10 and 20 µM) for 1 hour and then treated with LPS for 1 hour. NF-κB protein samples were analyzed by western blot with specific antibodies. β-actin was used as a control. The values presented are the means ± SEM of three independent experiments, ^#^ *P* < 0.01 is significantly different from the control group; ^*^ *P* < 0.05 and ^**^ *P* < 0.01 are significantly different from the LPS group.

**Figure 9:**
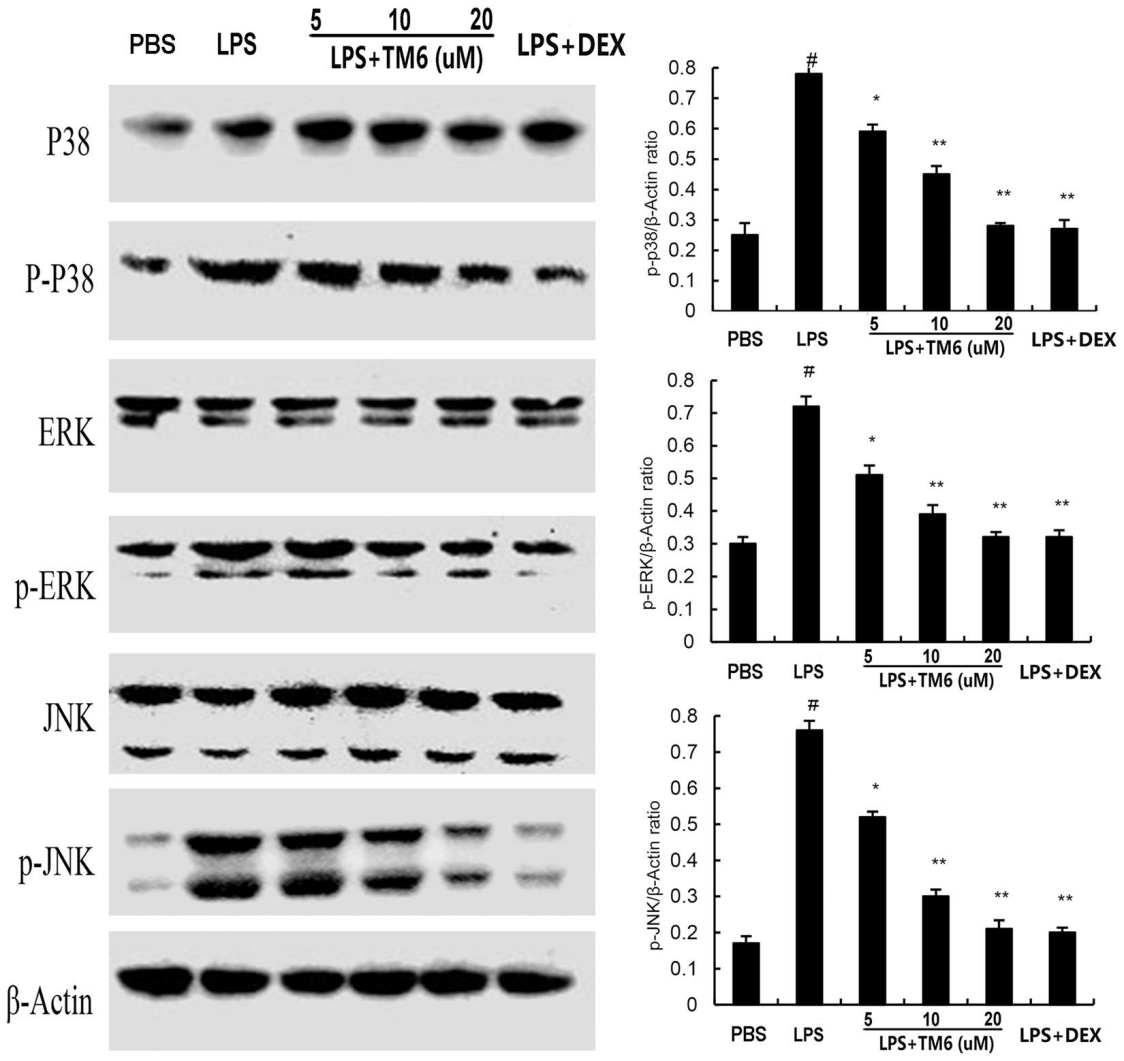
The effect of TM6 on MAPK pathway. Alveolar macrophages were pretreated with TM6 (5, 10 and 20 µM) for 1 hour and then treated with LPS for 1 hour. MAPK protein samples were analyzed by western blot with specific antibodies. β-actin was used as a control. The values presented are the means ± SEM of three independent experiments, ^#^ *P* < 0.01 is significantly different from the control group; ^*^ *P* < 0.05 and ^**^ *P* < 0.01 are significantly different from the LPS group.

To investigate the- protective mechanisms by which TM6 inhibits inflammatory cytokines production in alveolar macrophages, we detected NF-κB and MAPK signaling pathways by western blot analysis. The results showed that expression of p-IκB-α, p-p65, p-p38, p-ERK, and p-JNK increased significantly after LPS stimulation. However, TM6 (5, 10 and 20 µM) dose-dependently inhibited the expression p-IκB-α, p-p65, p-p38, p-ERK, and p-JNK induced by LPS (Figs 8 and 9).

## Discussion

Acute lung injury (ALI), a frequent complication of sepsis, can be caused by many factors, such as sepsis, trauma and bacterial pneumonia^18^. The development of ALI is associated with a large number of inflammatory cells migrating to the lung. Stimulation of these inflammatory cells lead to the releasing of inflammatory mediators. These inflammatory mediators disrupt pulmonary endothelial cells, pulmonary epithelial integrity and finally leads to lung edema^19, 20^. Corticosteroids, including DEX, triamcinolone acetonide, flunisolide and so on, have been widely used to treat inflammatory diseases like acute lung injury. Previous studies reported that treatment with DEX could improve the pathological process in ALI^21^. However, clinical application had suggested that these agents had local or systemic side effects. The reports have been suggested that some cell-permeable peptide can inhibited the development of inflammation *in vivo* and *vitro* ^12, 13^. Our previously studies also demonstrated that the peptide derived from TRAM or TIRAP also protected the development of mastitis induced by LPS in mice^14, 15^. In our study, we synthesized a cell-permeable TIR domain of TRAM-derived decoy peptide (TM6) and tested the protective effect of TM6 on LPS-induced ALI in mice. The results showed that the administration of TM6 markedly decreased lung wet/dry ratios, inflammatory cells production, pro-inflammatory cytokines production, and MPO activity. Moreover, TM6 significantly alleviated LPS-induced lung histopathological changes. *In vitro*, TM6 also inhibited the production of TNF-α, IL-1β and IL-6 and the expression of NF-κB and MAPK. For further research, we also tested the effect of intranasally administered of TM6 in LPS induced ALI. The results suggested that TM6 also inhibited the production of inflammatory cells and pro-inflammation cytokines in BLAF, as well as MPO activity in lung tissues. But the protective effect is not as effective compared with TM6 administered by intraperitoneal injection (data not shown). These findings suggested that TM6 may have potential value as a future therapeutic treatment to prevent ALI.

It is well known that edema is a typical symptom of ALI. Endothelial injury associated with microvascular leakage, was believed to be the main contributor of lung edema^19^. To analyze the magnitude of lung edema, the lung W/D ratio was measured. The results showed that administration of TM6 apparently decreased the lung W/D ratio, which suggested it suppressed the accumulation of serous fluid in lung tissues. From analyzing the lung pathology sections with light microscopy, we also found a resolution in pulmonary edema and most of the histopathological changes. MPO, the influx of a marker of neutrophil into the tissue, has been associated with tissue damage in many diseases^22^. In this study, our results showed that TM6 significantly inhibited LPS-induced MPO activity. These results suggested that TM6 had the ability to protect against LPS-induced ALI.

LPS has been reported to be one of the major factors that induces inflammatory response^23, 24^. In our model of ALI, LPS significantly increased the production of inflammatory cytokines including TNF-α, IL-1β and IL-6, which has been shown to be involved in the development of ALI^25, 26^. TNF-α is a primary pro-inflammatory factor, and is produced initially during infection^27^. IL-1β, another important cytokine, is one of the crucial mediators in ALI. Moreover, IL-1β is involved in the process of LPS-induced endotoxic shock and caused multiple organ failure^28^. IL-6, another pro-inflammatory cytokine, was considered to be a marker in the LPS-induced inflammatory response^27^. In this study, we measured the production of the cytokines TNF-α, IL-1β and IL-6, both *in vivo* and *vitro*. The results showed that TM6 significantly inhibited LPS-induced inflammatory cytokine production both *in vivo* and *in vitro*. These results indicated that TM6 has anti-inflammatory property to inhibit the production of cytokines during LPS-induced ALI.

NF-κB and MAPK, served as the major transcription factors, have been reported to play vital roles in the regulation of inflammatory cytokines production^29, 30^. Generally, once stimulated, NF-κB activation is initiated by the IκB-α and then NF-κB p65 is translocated to the nucleus. This process may promote the transcription of TNF-α, IL-1β, and IL-6^31^. MAPK family proteins including p38, JNK and ERK, all participate in the production of inflammatory cytokines^32^. To investigate the anti-inflammatory mechanism of TM6 on LPS-induced ALI, we determined the effects of TM6 on NF-κB and MAPK activation. The results showed that LPS-induced NF-κB and MAPK activation were markedly blocked by administration of TM6.

In conclusion, our study showed that TM6 was able to decrease inflammatory cell infiltration into the lung tissues, lung W/D ratio, MPO activity, inflammatory cytokines production, and lung histopathological changes. Moreover, the protective effect of TM6 may be related to attenuation of inflammatory reactions and inhibition of the activation of NF-κB and MAPK signaling pathways. These findings provide powerful evidence that TM6 may be a potential treatment for preventing ALI induced by LPS.

## Materials and Methods

### Animals

Adult male Balb/c mice were provided by the Experimental Animal Center of Baiqiuen Medical College of Jilin University (Changchun, China). The procedures were approved by the Jilin University Animal Care and Use Committee. The mice were supplied with food and water in a constant environment with the temperature at approximately 24 ± 1 °C and 40% to 80% relative humidity. All experiments followed the regulations for the Use and Care of Laboratory Animals Manual published by the US National Institutes of Health.

### Reagents

TM6 (Purity > 95%) was purchased from and synthesized by SBS Genetech Co. (Beijing, China). LPS (Escherichia coli 055:B5) was obtained from Sigma (St. Louis, MO, USA). Dexamethasone (Purity > 99%) was provided by Changle Pharmaceutical Co. (Xinxiang, Henan, China). ELISA kits were purchased from Biolegend (San Diego, CA, USA). Phospho-IκBα and, phospho-NF-κB p65 antibodies were purchased from Cell Signaling Technology Inc. (Beverly, MA). All other chemicals were reagent grade.

### ALI model

Mice were randomly divided into six groups: a control group, an LPS group, TM6 (2.5, 5 and 10 nmol/g) + LPS groups, and a DEX (5 mg/kg) + LPS group. The mice of TM6 (2.5, 5 and 10 nmol/g) + LPS groups were received an intraperitoneal (i.p.) injection of TM6 (2.5, 5 and 10 nmol/g) 1 h before LPS treatment. The mice of DEX + LPS group (5 mg/kg DEX) were received i.p. injections using the same method to avoid confounding procedural differences between groups. An equal volume of PBS, instead of TM6 or DEX, was given to the LPS group and control group. Then, 1 h later, 10 µg of LPS in 50 µl of PBS was introduced intranasally (i.n.) to produce acute lung injury. Control mice received 50 µl of PBS. All mice were humanely sacrificed 7 h after LPS treatment. Then, the bronchoalveolar lavage fluid (BALF) were collected for subsequent analysis.

### Histopathological evaluation

Histopathological analysis proceeded via H & E staining and light microscopy. Lung tissues were fully fixed with 10% buffered formalin for approximately 1 week, followed by paraffin-embedding and eosin staining. Histopathological changes of the lungs were examined with a light microscope (Olympus, Japan). The scores of histological changes in the lungs were evaluated as previously described^33^. Each animal is the average score of the above four criteria, including thickening of alveolar walls and epithelium, the numbers of infiltration cell, increase in peribronchial, and perivascular cuff area. The histological changes were scored 0 to 5.

### Lung wet/dry ratio

The lung lobe tissues were extracted and weighted to obtain the wet weight. Then, the tissues were incubated in 80 °C for 48 h to obtain the dry weight. Finally, the lung W/D ratio was calculated.

### Inflammatory cell numbers of BALF

BALF was collected 7 h after ALI was induced by LPS. After centrifugation (3000 rpm at 4 °C) for 10 min, the supernatants were collected and used to analyze the production of inflammatory cells.

### Pulmonary myeloperoxidase activity analysis

MPO activity reflects accumulation of macrophages and neutrophils in the lung tissues. The MPO activity in the lung tissues was measured by MPO kit according to the manufacturer’s instructions.

### Cell culture and treatment

Mice alveolar macrophages were isolated and cultured as previously described^34^. In brief, the mice were killed and the lungs were lavaged with PBS. The fluids were collected and centrifuged at 1000 g for 10 min at 4 °C. Then, the supernatants were removed and the cells were resuspended with RPMI 1640 media. The cells were plated in well plate and cultured at 37 °C in 5% CO_2_ incubator for 4 hours. Then the cells were washed once a day for 3 days. The nonadherent cells were removed and attached cells were cultured in RPMI 1640 media with 10% fetal calf serum. The media was changed once every day. In the study, the mice alveolar macrophages were preincubated with or without TM6 (5, 10 and 20 µM) for 1 hour before stimulation with LPS (1 µg/ml).

### Cell viability assays

Cell viability was tested by MTT assay. The alveolar macrophages were plated at a density of 4 × 10^5^ cells/ml in 96-well plates at 37 °C. 1 hour later, the cells were treated with TM6 at the dose with 0–40 µM and then cultured for another 1 hour. Next, the cells were treatment with LPS for 18 hour, followed by treatment with MTT (5 mg/ml). The cells were incubated for an additional 4 hours. Then, the supernatants were removed and DMSO (150 µl/well) were added to each well. The OD was read at 570 nm on a microplate reader.

### Inflammatory cytokine assays

BALF was collected and centrifuged (3000 rpm, 4 °C) for 20 min. Supernatant samples were used to analyze the production of TNF-α, IL-1β and IL-6 using special ELISA kits according to the instructions provided by the manufacturer.

### Western blot analysis

The proteins from the alveolar macrophages were extracted with mammal protein extraction reagent (M-PER) according to the manufacturer’s instructions. Equal amounts of protein (30 µg) were subjected to SDS-PAGE on 10% gels and transferred to PVDF membranes. The membranes were blocked with 3% BSA at room temperature on a rotary shaker for 2 h. Incubation with specific primary antibodies (1:1000) in TBS-T was performed overnight at 4 °C. Following washing, the membranes were incubated with secondary antibody for 1 h. The proteins bands were detected using an enhanced chemiluminescence western blotting detection kit.

### Statistical analyses

All values are expressed as the means ± SEM. Differences between groups were analyzed using a one-way ANOVA (Dunnett’s t-test) and a two-tailed Student’s t-test. The results were considered statistically significant at p < 0.05 or p < 0.01.
